# Impacts of an egg complementary feeding trial on energy intake and dietary diversity in Malawi

**DOI:** 10.1111/mcn.13055

**Published:** 2020-07-20

**Authors:** Chessa K. Lutter, Bess L. Caswell, Charles D. Arnold, Lora L. Iannotti, Kenneth Maleta, Raphael Chipatala, Elizabeth L. Prado, Christine P. Stewart

**Affiliations:** ^1^ RTI International, Washington, DC, School of Public Health University of Maryland College Park Maryland USA; ^2^ Department of Nutrition University of California Davis Davis California USA; ^3^ Brown School, Institute for Public Health Washington University in St. Louis St. Louis Missouri USA; ^4^ School of Public Health and Family Medicine University of Malawi College of Medicine Blantyre Malawi

**Keywords:** childhood diet, complementary feeding, complementary foods, dietary intake assessment, infant and child nutrition, randomized controlled trial

## Abstract

Complementary feeding diets in low‐ and middle‐income countries are generally inadequate to meet requirements for growth and development. Food‐based interventions may prevent nutrient inadequacies provided that they do not displace other nutrient‐rich foods. We conducted a randomized controlled trial in rural Malawi in which 660 children aged 6 to 9 months were provided an egg a day for 6 months or assigned to a control group. Dietary intake of complementary foods and drinks was assessed at baseline, 3‐month midline and 6‐month endline visits using a tablet‐based multipass 24‐h recall. Up to two repeat recalls were collected at each time point in a subsample of 100 children per treatment group. At midline and endline, usual energy intake from eggs was about 30 kcal/day higher in the egg group compared with controls (*p* < 0.0001). Compared with controls, children in the egg group were over nine times more likely to consume eggs at midline and endline. There was a comparable, but nonsignificant, greater total usual energy intake from complementary foods of 30 kcal/day at midline (*p* = 0.128) and 36 kcal/day at endline (*p* = 0.087). There also was a displacement of 7 kcal/day in legumes and nuts in children at endline (*p* = 0.059). At midline and endline, more than 80% of children in the egg group consumed a minimally diverse diet compared with 53% at midline and 60% at endline in the control group. This study illustrates that mothers in the egg group fed eggs to young children on a regular basis without substantial displacement of other complementary foods.

**Key messages**

- In rural Malawi, complementary feeding diets are of poor nutritional quality with the majority of calories from grains, roots and tubers—particularly maize—and very little consumption of animal‐source foods.
- An egg intervention early in complementary feeding was associated with large increases in consumption, illustrating that mothers are amenable to feeding eggs to young children without substantially displacing other foods from the children's diets. The intervention resulted in higher dietary diversity and increased percentage of children meeting a minimum dietary diversity cut‐off.
- Improving young children's diets should be viewed as an important outcome in and of itself, regardless of other nutrition outcomes.

## 1 INTRODUCTION

Adequate nutrition during infancy and early childhood is fundamental for children to reach their full human potential (Black et al., 2013). The World Health Organization (WHO, 2003) recommends that infants be exclusively breastfed for the first 6 months of life and at 6 months receive complementary foods that are adequate, safe and properly fed while breastfeeding continues for 2 years or more. The Guiding Principles for Complementary Feeding of the Breast‐fed Child and Guiding Principles for Feeding Non‐breastfed Children 6–23 Months of Age recommend including meat, poultry, fish or eggs as often as possible (PAHO/WHO, 2003; WHO, 2005). Complementary feeding diets in low‐ and middle‐income countries are generally of poor nutritional quality (Gibson, Ferguson, & Lehrfeld, 1998). Animal‐source foods (ASFs) including milk, meat, fish and eggs provide essential nutrients in highly bioavailable forms, yet are not consumed or are consumed in small amounts (Iannotti, 2018). Among these, eggs are relatively less expensive and potentially more affordable to low‐resource households (Iannotti, Lutter, Bunn, & Stewart, 2014).

Food‐based interventions have the potential to improve nutrient intake and growth in young children as long as they do not replace breast milk or nutrient‐dense foods from the diet (Arimond et al., 2015). Infants and young children 6 to 24 months who are breastfed and consume an average amount of breast milk need only between 200 and 550 kcal/day to satisfy their energy requirements (Dewey & Brown, 2003). To avoid displacement of breast milk, nutrient‐dense foods that do not provide excessive energy should be provided. A single 60‐g egg provides about 72 kcal and is packed with high‐quality protein and micronutrients and minerals supportive of child growth and development (Iannotti et al., 2014).

Young children need to consume a variety of foods to ensure that nutrient needs are met to support healthy growth and development. A diet lacking in diversity increases the risk of micronutrient deficiencies; however, consuming a diverse diet is important for reasons beyond meeting nutrient requirements. Foods are extraordinarily complex and include components, in addition to nutrients, that are biologically active and have health promoting effects (Jacobs & Tapsell, 2007, 2013). This is especially true of eggs (Iannotti et al., 2014).

The Lulun Project, a 6‐month egg intervention early in complementary feeding in the Ecuadorian Andes, resulted in 47% reduction in the prevalence of stunting and increase in biomarkers associated with cognitive development (Iannotti, Lutter, Stewart, et al., 2017; Iannotti, Lutter, Waters, et al., 2017). Similar to the Lulun Project, the Mazira Project sought to evaluate the effect of daily consumption of eggs over 6 months on the growth of children aged 6 to 9 months at baseline but, in contrast to the Lulun Project, did not observe a difference in child growth (Stewart et al., 2019). Here, we describe the contribution of daily egg supplementation to usual energy intake, usual energy intake by food group and minimum dietary diversity (MDD) of rural Malawian children. We hypothesize that (1) usual energy intake, excluding energy from breast milk, is higher among children in the egg group than in the control group at midline (3 months after baseline) and endline (6 months after baseline); (2) eggs consumed by the egg group do not displace usual energy intake from non‐egg foods, in total or by food group, at midline and endline; and (3) the dietary diversity score and prevalence of MDD, defined by WHO and partners (2008), are higher among children in the egg group than in the control group at midline and endline.

## 2 METHODS

### 2.1 Study design, participants and sample size

We describe details of the study elsewhere (Stewart et al., 2019). Briefly, we conducted a controlled trial, randomized by individual and carried out in rural areas in Mangochi District, Malawi. Infants aged 6.0–9.9 months were eligible to participate if they lived in the catchment area, were a singleton child and had a mid‐upper arm circumference >12.5 cm and no evidence of bipedal oedema, haemoglobin concentration >5 g/dl, no acute illness or injury requiring hospital referral, no history of egg allergy and no reaction to egg during a test feeding, no history of anaphylaxis or severe allergic reaction to any substance, no congenital defects or chronic morbidity associated with growth or development or that may affect feeding and no plans to leave the area in the next 6 months. Mothers of eligible children participated in a group information session during which the study was described in detail and one‐on‐one informed consent process. Mothers who agreed to participate were asked to sign or thumbprint the consent form and informed of their right to withdraw from the study at any time. All protocols were approved by the institutional review boards at the University of California, Davis, and the College of Medicine, Malawi.

Baseline data collection took place at the study clinic. Child length and weight were measured by trained anthropometrists using a length board (Harpenden Infantometer, Holtain Limited, UK) and digital flat scale for mother‐and‐baby weighing (Seca 874, Seca North America). Stunting, underweight and wasting were defined as *z*‐score <2 standard deviations (SDs) below the mean length‐for‐age, weight‐for‐age and weight‐for‐height, respectively, using the WHO child growth standards (WHO Multicentre Growth Reference Study Group, 2006). Malaria infection was determined by rapid diagnostic test (SD BIOLINE Malaria Ag P.f/Pan, Abbott Diagnostics, USA). Anaemia was defined as haemoglobin concentration <11 g/dl, as measured by haemoglobinometer (Hemocue 201, Hemocue Inc., Sweden). Demographic and socio‐economic data were collected by interview with the mother. Additional data on household characteristics and food security, assessed using the Household Food Insecurity Access Scale (Coates, Swindale, & Bilinsky, 2007), were collected by interview with the mother during a home visit the week following enrolment.

Randomization occurred at the study clinic visit after initial data collection. Mothers selected and opened one opaque envelope to reveal the intervention group. The intervention consisted of one egg per day for the study child for 6 months. Eggs were delivered to the household twice‐weekly, and mothers were encouraged to give the egg to the study child only. To reduce the probability that the egg was shared, intervention households were provided an additional egg per day. Staff who conducted assessments were blinded to group assignment. We enrolled infants between February and July 2018, with follow‐up visits occurring during the subsequent 6 months. At 3 months, a midline visit including dietary and anthropometric assessments was conducted at participants' homes. At 6 months, participants returned to the study clinic for endline data collection. Our sample size of 331 children per group was calculated for the main trail outcome of linear growth and not for dietary differences associated with the intervention. For this secondary analysis, we are powered to detect a mean difference effect size of 0.22 SDs between groups (*α* = 0.05 and 1 − *β* = 0.80) for continuous outcomes using the 660 randomized participants.

### 2.2 Assessment of dietary intake

Dietary intake of complementary foods and drinks was assessed using a tablet‐based multipass 24‐h dietary recall (Caswell et al., 2019). In a subsample of 100 children per group, replicate recalls were conducted on up to two additional days for each visit time point. Mothers were asked if they were breastfeeding, though breast milk intake was not assessed.

Interviewers for dietary data collection were trained over a 2‐week period on 24‐h interview technique, data recording and measurement of portion sizes. Supervisors completed shadow visits following a quality control checklist to ensure data quality over the course of the study. During the 24‐h recall, the child's mother was asked to recall all foods and drinks her child consumed between waking on the previous day and waking on the day of the recall. In the first pass, the mother was asked to report all foods consumed. In the second pass, she was asked for a detailed description of each food, the ingredients included in mixed dishes and the time her child consumed the food. In the third pass, she was asked to demonstrate the portion size of each food or drink her child consumed. Portion sizes were demonstrated using rice, modelling dough or water, according to the texture of the food or drink as served, as described by Gibson and Ferguson (2008). The mother was asked to show any portion left over, which was excluded from the estimated portion size. The interviewer measured the rice, dough or water using a 250‐ or 500‐ml graduated cylinder. In the final pass, the interviewer reviewed the information recorded in the first three passes and checked for any missed foods. The 24‐h recall interview also included questions on current breastfeeding status, use of supplements and factors that may have affected food intake.

We converted dietary data to observed energy intakes as follows:

1. Portion sizes were converted to grams using locally collected data on food densities, supplemented with food density data from the Nutrition Data System for Research (NDSR), US Department of Agriculture (USDA, 2018) and the Food and Agriculture Organization of the United Nations (FAO) (Charrondiere, Haytowitz, & Stadlmayr, 2012; Schakel, 2001).
2. Mixed dishes were disaggregated into ingredients using recipe data collected via focus groups with local women, as described by Gibson and Ferguson (2008).
3. Energy intake from each individual food/ingredient was estimated using food composition data from NDSR, supplemented with data from the USDA and regional food composition tables (Campbell et al., 2018; Hemsworth et al., 2016; Hotz & Gibson, 2001; Mengistu, Moges, Samuel, & Baye, 2017; Thakwalakwa et al., 2015). The energy content of eggs was estimated by laboratory analysis (Eurofins Scientific Inc., Des Moines, Iowa) of locally purchased eggs from the same commercial producer as used in the study.
4. Energy intakes from individual foods were summed to obtain the observed energy intakes from complementary foods in total and by food group as defined below.

### 2.3 Definition of outcome variables

#### 2.3.1 Usual energy intake

We estimated the distribution of usual energy intakes from foods and drinks in total and by food group using the National Cancer Institute (NCI, 2012) approach (Tooze et al., 2010) detailed below. This method adjusts for measurement error—primarily from day‐to‐day variability in intakes—in observed, single‐day estimates of nutrient intakes using repeat recalls.

#### 2.3.2 Dietary diversity

To describe dietary diversity, we used food groups to assess infant and young child feeding practices as defined by WHO and partners (2008): (1) grains, roots and tubers; (2) legumes and nuts; (3) dairy products (milk, yogurt and cheese); (4) flesh foods (meat, fish, poultry and liver/organ meats); (5) eggs; (6) vitamin A‐rich fruits and vegetables; and (7) other fruits and vegetables. We also created an eighth category of snack foods and sugar‐sweetened beverages as described in Campbell et al. (2018). Any foods not falling in these eight predefined food groups were categorized as ‘other’. For the dietary diversity analyses, mixed dishes were disaggregated, and each ingredient was separately assigned to its corresponding food group. MDD was determined by consuming four or more of the seven food groups, excluding the additional category of snacks and sugar‐sweetened beverages and other, uncategorized foods.

### 2.4 Statistical analyses

All testing was two‐sided and considered significant at the *α* = 0.05 level, unless otherwise specified. We used SAS version 9.4 primarily for analyses and results preparation and Stata version 14.1 primarily for data cleaning and management. Repeat recalls were excluded at baseline if they were not completed within 21 days after enrolment and prior to the first intervention and monitoring home visit; at midline if they were not completed within 10 days before or after the midline home visit; and at endline if they were not completed within 21 days before the endline clinic visit. As a result of these exclusion criteria, the number of children with repeat recalls is less than 100. A detailed statistical analysis plan was developed prior to initiating the analysis and posted publicly.

#### 2.4.1 Descriptive analyses

We used descriptive statistics to explore child, maternal and household characteristics by intervention group and between those with and without endline data. To describe dietary patterns, we calculated the frequency of consumption and the quantity per serving (grams) for the following categories of foods: nsima (the local staple dish of stiff maize porridge); phala (soft porridge, typically prepared only for young children); fish; eggs; meat; dairy; fruit; green leafy vegetables; orange fleshy vegetables; other vegetables; potatoes, rice or pasta; sweets; savoury snacks; legumes; juice; tea; bread; and other foods. For this analysis, mixed dishes were categorized by main ingredient and, for maize‐based dishes, preparation method.

#### 2.4.2 Difference in usual energy intakes and dietary diversity by intervention group

To test the impact of the intervention on usual energy intake from foods and usual energy intake by food group, we used the NCI (2012) macros with bootstrap standard errors (National Cancer Institute and National Center for Health Statistics, 2014) to estimate usual intake distributions by intervention group (Tooze et al., 2010). The MIXTRAN macro fits a mixed effects model of usual energy intake, and the DISTRIB macro uses a Monte Carlo procedure to estimate mean and percentiles of the usual intake distribution. We included child age, sex, report of illness in the previous 24 h, report of unusual food intake during the recall period and whether the recall day was a market day as potential fixed effects on total energy intake or energy intake by food group. Unusual food intake and report of illness were only included in the final usual intake models if they showed a bivariate association (*p* < 0.1) with the outcome variable.

Usual intake distributions were modelled separately by group to produce estimates of mean usual intake that appropriately allow day‐to‐day intake variation to be different in the two groups. For each variable describing usual energy intake or usual energy intake by food group, we repeated the usual nutrient intake modelling procedure 200 times, using bootstrap samples created by random draw with replacement. Usual intake was estimated for total energy, the seven WHO food categories, total energy without eggs and snack foods. Although the above were pre‐specified, we subsequently added an ‘other’ category to capture the full diet. This category consists primarily of energy from oil consumption. We used the original sample point estimates of usual intake and bootstrap standard error estimates to construct 95% confidence intervals (CIs) and calculate *p*‐values for difference in mean by intervention group based on unequal variance *t*‐tests.

We tested for differences in mean dietary diversity score using negative binomial regression and for changes in prevalence of MDD using log‐binomial regression. All models controlled for baseline status and adjusted models controlled for child age, sex and report of illness or change in appetite if marginally associated with the outcome in bivariate regressions (*p* < 0.1). In addition, we similarly compared the percentages of children in each group who consumed foods from each of the seven WHO food groups at midline and at endline. Statistical analysis plans were determined prior to any analyses and posted at https://osf.io/vfrg7/ .

## 3 RESULTS

**FIGURE 1:**
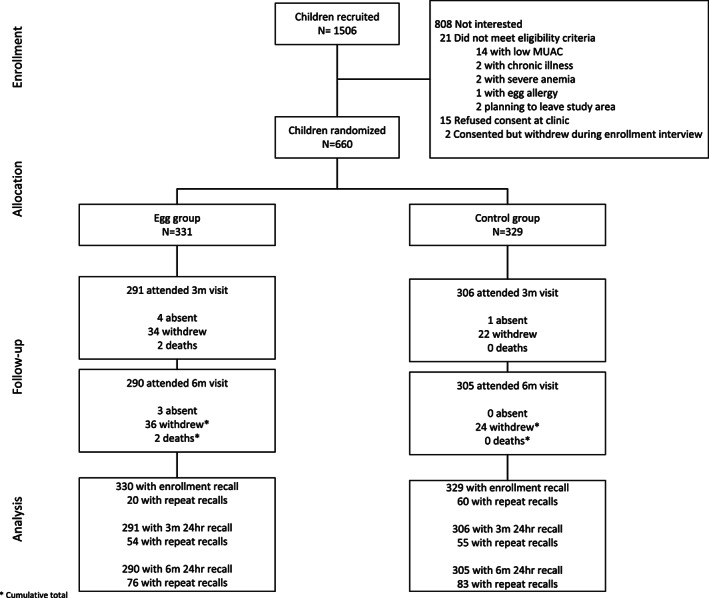
Participant flow diagram. MUAC, mid‐upper arm circumference

A total of 331 and 329 infants were randomized to the egg and control groups, respectively (Figure 1). All have baseline dietary data. In the egg group, dietary data are available for 291 children at the 3‐month visit (midline) and 290 children at the 6‐month visit (endline), and in the control group, dietary data are available for 306 children at the 3‐month visit and 305 children at the 6‐month visit. The overall loss to follow‐up was 12% in the egg group compared with the control group (7%). A total of 60 children withdrew, three were absent, and two died. Compared with those who were followed, mothers of children who withdrew were less likely to report being able to read and more likely to be food insecure. All other characteristics of those who completed the study or withdrew were generally similar.

There were no statistically significant differences between the intervention groups in baseline characteristics (Table 1). The mean age of children was 7.3 months in the control group and 7.4 months in the egg group, and about a quarter were firstborn. Less than half of mothers were able to read, and less than one quarter had completed primary schooling, with the proportion who completed primary school lower in the control group (16%) compared with the egg group (24%). The prevalence of anaemia and malaria in children was 60% and 13%, respectively. Just over 80% of mothers in the control group reported moderate or severe household food insecurity compared with 75% of mothers in the egg group. In both groups, 88% of households were Muslim. At baseline, all but one child was breastfed, and breastfeeding continued throughout the study with very few exceptions. Usual energy intake from complementary foods at baseline was similar between groups. The prevalence of stunting and underweight was around 13% and 8%, respectively. There was virtually no wasting.

**TABLE 1:** Enrolment characteristics by treatment group

Characteristics	Control		Egg	
	*N*	% or mean (SD)	*N*	% or mean (SD)
Maternal				
Maternal age (years)	325	26.1 (6.8)	329	25.9 (6.7)
Maternal BMI (kg/m^2^)	329	21.8 (2.8)	331	21.8 (3.2)
Maternal education				
Completed primary or greater	329	16.4	331	23.6
Mother can read	321	41.7	322	50.0
Maternal marital status				
Monogamous	329	59.9	331	55.6
Polygamous		21.3		18.7
Unmarried		18.8		25.7
Child				
Child age (months)	329	7.3 (1.2)	331	7.4 (1.2)
Female	329	48.3	331	48.3
Firstborn	329	25.2	330	30.0
Malaria	296	12.5	299	12.7
Anaemia	290	61.4	292	59.9
Breastfeeding	329	100.0	330	99.7
Usual energy intake^a^ (kcal)	329	259 ± 9	330	273 ± 9
Household				
Muslim	321	88.2	322	87.9
Number of children under 5 years	319	1.7 (0.8)	319	1.7 (0.8)
Number of household members	320	6.0 (2.7)	321	5.8 (2.6)
Moderate or severe food insecurity^b^	329	81.2	331	74.6
Own latrine	321	96.6	322	96.3
Distance to water source				
<10 min	321	54.5	322	56.8
Any chickens owned	329	35.6	330	29.1
Anthropometry				
Stunted	329	14.0	331	13.3
Underweight	329	8.5	331	7.3
Wasted	329	1.2	331	0.9

*Note*: % or mean (standard deviation [SD]).

Abbreviation: BMI, body mass index.

^a^ National Cancer Institute estimate of usual energy intake from complementary foods.

^b^ Food insecurity assessed using the Household Food Insecurity Access Scale (Coates et al., 2007).

At baseline, patterns of food consumption of children were similar between groups (Table 2). Nearly all consumed phala (soft maize porridge), with a median intake of about 115 g. About 55% consumed nsima (stiff maize porridge), with consumers having an average portion size of 60 g. Savoury snacks were the third most common category of food—predominantly in the form of Kamba Puffs, a maize‐based extruded snack—and were consumed by 30% of children who consumed on average about 6 g. Green leafy vegetables, such as pumpkin leaves, were the fourth most commonly consumed food and consumed by about a quarter of children. Fish was the next most commonly consumed food, consumed by 22% and 32% of children in the control and egg groups, respectively. Among children who consumed fish dishes, the mean intake was 23 g in the control group and 20 g in the egg group. Fish was followed by legumes such as red beans and cow peas. Sweets, primarily in the form of hard candy, were consumed by about 8% of children. Eggs, meat and dairy were rarely consumed, as were foods in the other groups.

**TABLE 2:** Baseline food composition by recipe type and primary ingredient

Food	*N* (%)^a^	Amount (g)	*N* (%)^a^	Amount (g)
Phala (porridge)	314 (96.0)	115.8 [87.2, 158.2]	322 (97.6)	112.0 [79.1, 158.2]
Nsima	179 (54.7)	61.5 [42.8, 85.6]	189 (57.3)	58.9 [42.8, 85.6]
Savoury snacks	100 (30.6)	6.3 [3.6, 10.8]	94 (28.5)	6.1 [3.6, 9.8]
Green leafy vegetables	82 (25.1)	20.4 [15.6, 30.0]	71 (21.5)	20.0 [15.0, 30.0]
Fish	72 (22.0)	22.7 [14.7, 30.6]	105 (31.8)	20.4 [14.4, 30.6]
Legumes	44 (13.5)	22.9 [15.0, 33.6]	42 (12.7)	21.8 [12.9, 32.7]
Tea	29 (8.9)	78.5 [51.0, 102.0]	23 (7.0)	61.2 [48.5, 81.6]
Sweets	25 (7.6)	17.6 [13.3, 41.5]	30 (9.1)	33.2 [14.7, 49.8]
Dairy	11 (3.4)	61.8 [41.2, 103.0]	15 (4.5)	52.5 [42.0, 153.0]
Fruit	11 (3.4)	43.6 [27.0, 60.6]	13 (3.9)	24.9 [16.0, 38.8]
Orange fleshy vegetables	8 (2.4)	36.7 [29.7, 52.3]	10 (3.0)	22.6 [15.6, 25.4]
Potatoes, rice or pasta	8 (2.4)	42.7 [21.9, 57.5]	15 (4.5)	31.1 [19.4, 56.5]
Eggs	7 (2.1)	27.4 [22.6, 40.2]	11 (3.3)	41.2 [27.9, 59.5]
Bread	7 (2.1)	5.1 [2.2, 10.2]	4 (1.2)	6.8 [5.1, 8.5]
Juice	6 (1.8)	90.7 [58.1, 143.6]	2 (0.6)	106.0 [60.6, 116.6]
Meat	3 (0.9)	29.7 [26.0, 39.6]	7 (2.1)	26.0 [17.3, 32.2]
Other vegetables	1 (0.3)	15.3 [12.9, 21.4]	1 (0.3)	28.2 [19.4, 33.2]

*Note*: Values are median [Q1, Q3]. Amount is reported only among children who consumed food.

^a^ *N* and per cent of children who consumed food.

**FIGURE 2:**
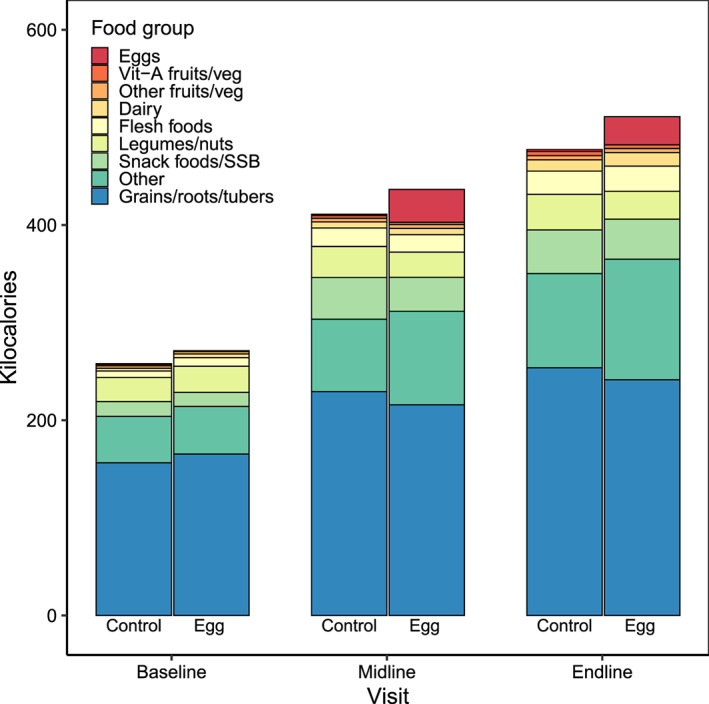
Usual energy intake by food group at baseline, midline and endline. SSB, sugar‐sweetened beverages

At baseline, usual intake of total energy from foods and energy from specific food groups did not differ between the control and egg groups (Figure 2). Usual daily energy intake was 259 kcal in infants in the control group and 273 kcal in the egg group. Grains, roots and tubers were the largest source of energy followed by the category ‘other’. This category consists of ingredients that are not otherwise caught by food group codes, such as cooking oil (59%), sugar (33%) and black tea (6%). Legumes and nuts were the next most commonly consumed food category, with the remaining categories contributing 15 kcal/day or less to usual energy intakes.

At midline, there was a 30‐g difference in usual energy intake from foods; the egg group consumed 440 kcal/day (95% CI 328, 439), and the control group consumed 410 kcal/day (95% CI 414, 466). At endline, there was a 36 kcal/day of difference in usual energy intake from foods; the egg group consumed 515 kcal/day (95% CI 485, 545), and the control group consumed 479 kcal/day (95% CI 451, 507) (Figure 2; Table S1). Usual energy intake without eggs did not differ between groups at midline or endline; children consumed on average just over 400 kcal/day of foods and drinks other than eggs at midline and about 480 kcal/day at endline. At midline, usual energy intake from eggs was significantly higher in the egg group (34 kcal/day; 95% CI 30, 38) compared with 1 kcal/day (95% CI 1, 2) in the control group (*p* < 0.001). At endline, usual energy intake from eggs in the egg group was slightly lower than at midline but was still significantly higher than in the control group. Intakes of foods categorized as ‘other foods’, such as oil and sugar, were significantly higher in the egg group at midline and endline. At midline, children in the egg group consumed 96 kcal (95% CI 84, 108) compared with 74 kcal in children in the control group (95% CI 64, 84). At endline, children in the egg group consumed 124 kcal (95% CI 112, 135) compared with 96 kcal in children in the control group (95% CI 87, 105). There were no other significant differences in usual energy intake between the two groups in other food categories except for legumes/nuts, which was marginally significantly lower by about 7 kcal/day in the egg group at endline (*p* = 0.06) though not at midline. Covariate adjusted analyses were similar and provided the same inference (data not shown).

At baseline, only about one in four children consumed a minimally diverse diet (Table 3). Dietary diversity increased in both groups at midline and endline, though compared with controls, increases were significantly greater in the egg group. At midline, children in the egg group were 57% more likely to achieve MDD compared with children in the control group (84% vs. 53%), and at endline, children in the egg group were 34% more likely to achieve MDD compared with children in the control group (80% vs. 60%). Except for eggs, consumption of foods in the other categories did not differ between groups. Children in the egg group were nearly 13 times and 10 times more likely to consume eggs compared with children in the control group at midline and endline, respectively. Covariate adjusted analyses were similar (data not shown), so final analyses included adjustment for baseline values only.

**TABLE 3:** Dietary diversity score, mean and prevalence ratio

	Baseline		Midline		Endline			
	Control	Egg	Control	Egg	Control	Egg	Minimally adjusted^a^ ratio at midline (95% CI)	Minimally adjusted^a^ ratio at endline (95% CI)
	*N* = 329	*N* = 330	*N* = 306	*N* = 291	*N* = 305	*N* = 290		
Seven‐group DDS	2.47 (1.24)	2.56 (1.30)	3.60 (1.11)	4.31 (1.08)	3.74 (1.13)	4.34 (1.10)	1.19 (1.10, 1.29)	1.16 (1.07, 1.25)
Minimum DDS^b^	21.6	25.2	52.9	83.8	59.7	80.3	1.57 (1.40, 1.77)	1.34 (1.20, 1.49)
Consumed grains/roots/tubers	98.5	99.1	98.7	99.3	98.7	99.3	‐	‐
Consumed legumes/nuts	35.6	35.5	47.1	45.7	49.2	43.8	0.97 (0.82, 1.15)	0.90 (0.76, 1.06)
Consumed dairy	8.5	6.4	10.8	9.3	19.7	17.6	0.95 (0.60, 1.51)	0.92 (0.66, 1.28)
Consumed flesh foods	23.4	33.6	68.0	65.3	66.9	72.1	0.96 (0.85, 1.07)	1.07 (0.96, 1.19)
Consumed eggs	4.0	4.2	6.5	84.2	7.2	70.7	12.86 (8.41, 19.69)	9.78 (6.50, 14.73)
Consumed vitamin A fruits/vegetables	26.4	24.5	39.2	36.4	41.6	38.3	0.94 (0.77, 1.15)	0.93 (0.76, 1.13)
Consumed other fruits/vegetables	50.5	52.7	89.5	91.1	90.5	92.4	1.02 (0.96, 1.07)	1.02 (0.97, 1.07)

*Note*: Values are mean (standard deviation) with mean ratio for the seven‐group DDS count outcome and prevalences with prevalence ratios for all other binary outcome comparisons.

Abbreviations: CI, confidence interval; DDS, dietary diversity score.

^a^ Adjusted for baseline value.

^b^ Minimum DDS defined as consuming four or more of the seven indicated food groups.

## 4 DISCUSSION

In a trial providing an egg a day to 6‐ to 15‐month‐old children, children in the egg group consumed a mean usual energy intake from eggs of 30 kcal/day compared with the control group mean usual intake of 1–2 kcal/day. Total usual energy consumption from complementary foods was also approximately 30 kcal/day higher in children in the egg group compared with the children in the control group at both midline and endline, though this difference was marginally significant. These results suggest an additive effective of the intervention of approximately half an egg per day. We did not collect data on what happened to the rest of the egg.

Virtually, all children were breastfed, and energy intake from breast milk was not measured. Energy requirements for infants and young children 6 to 23 months of age in low‐ and middle‐income countries are approximately 615 kcal/day for infants 6 to 8 months, 686 kcal/day for infants 9 to 11 months and 894 kcal/day for children 12 to 23 months (Food and Agricultural Organization, World Health Organization & United Nations University, 2004). Dewey and Brown estimated that breastfed children consuming an average amount of breast milk only need to consume an average of 200 kcal/day from complementary foods from 6 to 8 months, 300 kcal/day from 9 to 11 months and 550 kcal/day from 12 to 23 months (WHO, 1998). Assuming that children in our study consumed an average amount of breast milk and given that usual energy intake from complementary foods in our control group was on average 259 kcal/day at baseline, 410 kcal/day at midline and 479 kcal/day at endline, with even higher intakes in the egg group, both groups almost certainly met their energy requirements. Therefore, energy does not appear to be a limiting factor in their diets.

The intervention resulted in some displacement in the consumption of legumes and nuts in the egg group, with the egg group consuming about 7 kcal/day less than controls. This difference was only marginally significant at endline and nonsignificant at midline. Groundnut, soya or bean flour are commonly used to enrich or flavour porridge. Mothers in the egg group commonly mixed eggs into porridge and also commonly fried eggs in oil, causing an increase in energy intake from cooking oil. The observed displacement in legume and nut consumption, an important source of micronutrients, protein and fats, may be a result of mothers occasionally changing porridge ingredients as a result of the intervention. The displacement effect was very small (7 kcal/day) and made up by the nutrients provided by the increased egg intakes.

As shown in the recent Demographic and Health Study in Malawi, complementary feeding diets are of very low quality, particularly in rural areas (National Statistical Office [Malawi] & ICF International, 2017). In our study, diets were also of poor quality with only about 25% of infants in both groups achieving MDD at baseline. The egg intervention significantly raised dietary diversity scores in the egg group compared with the control group at midline and endline. At both time points, children in the egg group were much more likely to meet the MDD cut‐off set by the WHO and partners. These changes were driven by consumption of eggs, with only minor differences in the consumption of legumes and nuts, and indicate that the egg intervention had an additive effect rather than displacing other foods. An increase in MDD is expected as children grow older, and in the control group, the per cent achieving the MDD improved as children aged, increasing from 25% at baseline to 53% at midline and nearly 60% at endline. Although the proportion of children who met the MDD was greater in the egg group than the controls, it still fell short of 100%. This is not unexpected as the intervention only focused on one of the seven food groups assessed.

Despite large and highly significant increases in egg intake, little substitution of eggs for other nutrient‐rich foods and improvements in MDD in children in the egg group compared with controls, the intervention did not result in improvements in linear growth or stunting reduction (Stewart et al., 2019) as was observed in the Lulun Project in Ecuador (Iannotti, Lutter, Stewart, et al., 2017). This may be due to the high prevalence of fish consumption (60%), difference in staple food (maize vs. potato) and low baseline prevalence of stunting (13%) compared with that in Ecuador. Although egg consumption remained high throughout the study, it was lower at endline compared with midline, suggesting some decrease in adherence over the 6‐month intervention. However, dietary diversity remained high in the egg group throughout the study.

The low baseline prevalence of stunting in our study was surprising given a prevalence of 32% among control children 6 months of age in the iLiNS trial conducted in the same study areas 7 years earlier (Maleta et al., 2015). The most recent Malawi Demographic Survey reported a stunting prevalence of 39% in rural children less than 5 years (National Statistical Office [Malawi] & ICF International, 2017). Interestingly, our study site was also the one site in the iLiNS trials that did not show an effect of lipid‐based nutrient spreads on child growth, despite large increases in micronutrient and macronutrient intakes (Adu‐Afarwuah, Lartey, & Dewey, 2007; Hemsworth et al., 2016). In addition to a low baseline prevalence of stunting and high fish consumption, there may be other unexplained factors in this setting that prevent children from responding to nutritional interventions.

Two multisectoral interventions that provided inputs other than food had an effect on child egg consumption and growth. In Ethiopia, a behaviour change intervention delivered through interpersonal communication, agricultural activities, community mobilization and mass media showed a strong association between the agricultural activities and egg consumption, which resulted in increased child dietary diversity and linear growth (Kim et al., 2016). In Ghana, a package of inputs and training in poultry farming and home gardening and education in nutrition and care resulted in increases in child dietary diversity and egg consumption and improvements in linear growth in the intervention group compared with controls (Marquis et al., 2018). In contrast, in Bangladesh, a behaviour change intervention that did not provide food or other inputs resulted in large improvements in complementary feeding diets, including egg consumption, but no increases in linear growth (Menon et al., 2016).

Our study had some weaknesses; because of the difficulty of having a field worker in the home for lengthy periods of time, daily egg consumption was assessed through maternal report rather than direct observation. Its strengths include a large sample size, few losses during follow‐up and large positive differences in egg consumption in the egg group compared with the control group. We also performed replicate 24‐h recalls at enrolment and follow‐up visits on a subsample of children to reduce measurement error due to day‐to‐day variation in diet.

The large increase in egg consumption in children in the egg group is additional proof that mothers are amenable to feeding eggs to young children as observed in the Ecuador study (Iannotti, Lutter, Stewart, et al., 2017; Walters et al., 2018). Although the systematic collection of cost data was not part of the study, anecdotally, we found that eggs were much more expensive than small fresh or dried fish. The finding supports the hypothesis that economic rather than cultural barriers are the main deterrent to improving egg consumption in children in low‐ and middle‐income countries (Headey & Alderman, 2019).

Our analysis adds detail to the emerging understanding of how a food‐based intervention can improve complementary feeding diets without displacing intakes of other nutrient‐rich foods and also increase dietary diversity in children during a critical period in their development. While stunting is frequently used as a measure of child well‐being, improving children's diets should be viewed as an important outcome in and of itself even if not translated to improvements in linear growth. ASFs are a critical source of high‐quality protein, vitamins and minerals essential for child growth and development, but economic barriers often stand in the way of families accessing these foods. More effort is needed to improve their access in low‐ and middle‐income countries.

## CONFLICTS OF INTEREST

The authors declare that they have no conflicts of interest.

## CONTRIBUTIONS

CPS, CKL, KM, ELP and CKL designed the research study. BLC, KM and RC performed the research. CKL, BLC and CDA analysed the data. CKL, BLC and CDA wrote the paper.

## Supporting information

Table S1. Usual energy intake at baseline, midline, and endline
