# Supplementary material for: Impacts of an egg complementary feeding trial on energy intake and dietary diversity in Malawi
Source: Matern Child Nutr. 2020 Jul 20;17(1):e13055. doi: 10.1111/mcn.13055 (PMC7729770; doi:10.1111/mcn.13055)
Supplement: Supplementary file 1 — Table S1. Usual energy intake at baseline, midline, and endline [file MCN-17-e13055-s001.docx]

Supplemental Table 1. Usual energy intake at baseline, midline, and endline among participants in the Mazira Project egg feeding trial (n=600), Mangochi District, Malawi, 2018-2019

|  |  |  |  | Consumers |
| --- | --- | --- | --- | --- |
| Food group | (95% CI) | (95% CI) | p-value^†^ | n (%) |
| **Baseline** | **Control (N=329)** | **Egg (N=330)** |  |  |
| Total energy | 259 (242, 277) | 273 (255, 291) | 0.275 | 657 (99.7) |
| Total energy without eggs | 259 (241, 276) | 272 (254, 290) | 0.289 | 657 (99.7) |
| Grains/roots/tubers | 156 (146, 167) | 165 (154, 177) | 0.253 | 651 (98.8) |
| Legumes/nuts | 25 (20, 29) | 27 (21, 33) | 0.593 | 234 (35.5) |
| Dairy | 3 (2, 4) | 4 (2, 6) | 0.426 | 49 (7.4) |
| Flesh foods | 7 (5, 8) | 9 (7, 10) | 0.059 | 188 (28.5) |
| Eggs | 1 (0, 1) | -^‡^ | -^‡^ | 27 (4.1) |
| Vit-A fruits/veg | 1 (1, 2) | 1 (1, 1) | 0.138 | 168 (25.5) |
| Other fruits/veg | 3 (2, 3) | 2 (2, 3) | 0.642 | 340 (51.6) |
| Snack foods/SSB | 15 (11, 19) | 14 (11, 18) | 0.773 | 227 (34.4) |
| Other foods | 47 (41, 54) | 49 (42, 55) | 0.800 | 456 (69.2) |
| **Midline** | **Control (N=306)** | **Egg (N=291)** |  |  |
| Total energy | 410 (382, 439) | 440 (414, 466) | 0.128 | 593 (99.3) |
| Total energy without eggs | 409 (381, 437) | 406 (381, 430) | 0.873 | 593 (99.3) |
| Grains/roots/tubers | 229 (216, 243) | 216 (202, 229) | 0.153 | 591 (99.0) |
| Legumes/nuts | 32 (26, 38) | 26 (21, 30) | 0.136 | 277 (46.4) |
| Dairy | 6 (1, 12) | 6 (1, 11) | 0.979 | 60 (10.1) |
| Flesh foods | 19 (17, 21) | 18 (16, 20) | 0.514 | 398 (66.7) |
| Eggs | 1 (1, 2) | 34 (30, 38) | 0.000 | 265 (44.4) |
| Vit-A fruits/veg | 3 (2, 4) | 2 (2, 3) | 0.441 | 226 (37.9) |
| Other fruits/veg | 4 (3, 4) | 4 (3, 4) | 0.618 | 539 (90.3) |
| Snack foods/SSB | 43 (33, 52) | 35 (27, 42) | 0.203 | 286 (47.9) |
| Other foods | 74 (64, 84) | 96 (84, 108) | 0.006 | 516 (86.4) |
| **Endline** | **Control (N=305)** | **Egg (N=291)** |  |  |
| Total energy | 479 (451, 507) | 515 (485, 545) | 0.087 | 590 (99.2) |
| Total energy without eggs | 477 (449, 505) | 486 (457, 516) | 0.663 | 589 (99.0) |
| Grains/roots/tubers | 254 (238, 270) | 241 (226, 257) | 0.271 | 589 (99.0) |
| Legumes/nuts | 36 (30, 43) | 29 (23, 34) | 0.059 | 277 (46.6) |
| Dairy | 12 (8, 15) | 14 (9, 18) | 0.425 | 111 (18.7) |
| Flesh foods | 24 (21, 26) | 26 (23, 29) | 0.287 | 413 (69.4) |
| Eggs | 2 (1, 3) | 29 (26, 32) | 0.000 | 227 (38.2) |
| Vit-A fruits/veg | 4 (3, 5) | 4 (3, 5) | 0.387 | 238 (40.0) |
| Other fruits/veg | 4 (4, 5) | 4 (4, 5) | 0.832 | 544 (91.4) |
| Snack foods/SSB | 45 (38, 51) | 41 (35, 47) | 0.402 | 381 (64.0) |
| Other foods | 96 (87, 105) | 124 (112, 135) | 0.000 | 547 (91.9) |

^†^Bootstrapped standard errors unequal variance t-test. Usual estimates are estimated separately

within each intervention group controlling for sex, age, and market day along with illness or

unusual consumption on the day of recall if associated with intake.

^‡^Models did not converge due to extreme outcome prevalence
